# Magnetoreception in Hymenoptera: importance for navigation

**DOI:** 10.1007/s10071-020-01431-x

**Published:** 2020-09-25

**Authors:** Pauline N. Fleischmann, Robin Grob, Wolfgang Rössler

**Affiliations:** Behavioral Physiology and Sociobiology (Zoology II), Biozentrum, University of Würzburg, Am Hubland, 97074 Würzburg, Germany

**Keywords:** Active sensing, *Cataglpyhis* desert ants, Honeybees, Learning walks, Magnetic compass, Path integration

## Abstract

The use of information provided by the geomagnetic field (GMF) for navigation is widespread across the animal kingdom. At the same time, the magnetic sense is one of the least understood senses. Here, we review evidence for magnetoreception in Hymenoptera. We focus on experiments aiming to shed light on the role of the GMF for navigation. Both honeybees and desert ants are well-studied experimental models for navigation, and both use the GMF for specific navigational tasks under certain conditions. *Cataglyphis* desert ants use the GMF as a compass cue for path integration during their initial learning walks to align their gaze directions towards the nest entrance. This represents the first example for the use of the GMF in an insect species for a genuine navigational task under natural conditions and with all other navigational cues available. We argue that the recently described magnetic compass in *Cataglyphis* opens up a new integrative approach to understand the mechanisms underlying magnetoreception in Hymenoptera on different biological levels.

## Introduction

Navigation by means of the earth’s magnetic field (or geomagnetic field, GMF) is one of the most impressive behavioral phenomena in the animal kingdom. Since, from a human perspective, introspection of the magnetic sense is lacking, the magnetic sense is the most difficult to comprehend from a cognitive perspective. This might be one reason why despite substantial research efforts—from pure observation via experimental manipulations and theoretical reflections—until now neither the location of the magnetic receptors nor the neuronal mechanisms underlying magnetoreception have been identified in any species and many aspects are controversial (Nordmann et al. 2017).

Since the first description of the existence of a magnetic compass in migratory birds in the second half of the twentieth century (Merkel and Wiltschko 1965), many more animal species have turned out to be magneto-sensitive, including many arthropods (for a review: Vacha 2017).

Already starting in the 1960s, less well-known, elegant experiments were performed with honeybees (Hymenoptera: Apidae: *Apis mellifera*), indicating that social insects belonging to the Hymenoptera can sense the GMF (Lindauer and Martin 1968). Since then many further experiments on hymenopteran insect species tried to shed light on the phenomenon of magnetoreception at different biological levels. In this position paper, we aim to compile and integrate the research efforts and progress made in hymenopteran magnetoreception within the past half century, especially by focusing on their implications for navigation.

Social Hymenoptera are especially well suited as experimental models for navigation, because they live with other colony members in their common nest where female workers collectively care for the queen (the only reproductive female) and the brood. Foraging workers search for food outdoors and subsequently have to return to the nest (central place foraging) to provide for the colony as a whole. Therefore, successful navigation during foraging and homing is essential not only for the individual, but also for the whole colony. We define “navigation” as a special case of spatial orientation during which an individual is constantly informed about its current position relative to its goal. One of the most important navigational mechanisms in Hymenoptera is path integration, during which an animal keeps track of distance and direction to return to the starting point by processing the information into a home vector (“beeline”) (e.g.,Wehner 1982; Collett and Collett 2000). The only experiments on Hymenoptera that were able to test the direct use of the GMF during navigation have been achieved using honeybees and ants as experimental models. By comparing the current evidence from research on different species within the Hymenoptera and using the strict definition of navigation, we argue that under natural conditions magnetoreception has proven to be crucial for at least one specific navigational task, the gazes back towards the nest entrance during initial learning walk pirouettes of the desert ant *Cataglyphis nodus* (Hymenoptera: Formicidae) (Fleischmann et al. 2018a). This does not mean that magnetoreception is not used in other hymenopteran species, but the very obvious use of a magnetic sense during close-range navigation in thermophilic *Cataglyphis* ants offers a highly promising experimental model that helps to unravel the mystery of magnetoreception, the “sense without a receptor” (Nordmann et al. 2017).

## The geomagnetic field (GMF) as a navigational cue

**Fig. 1:**
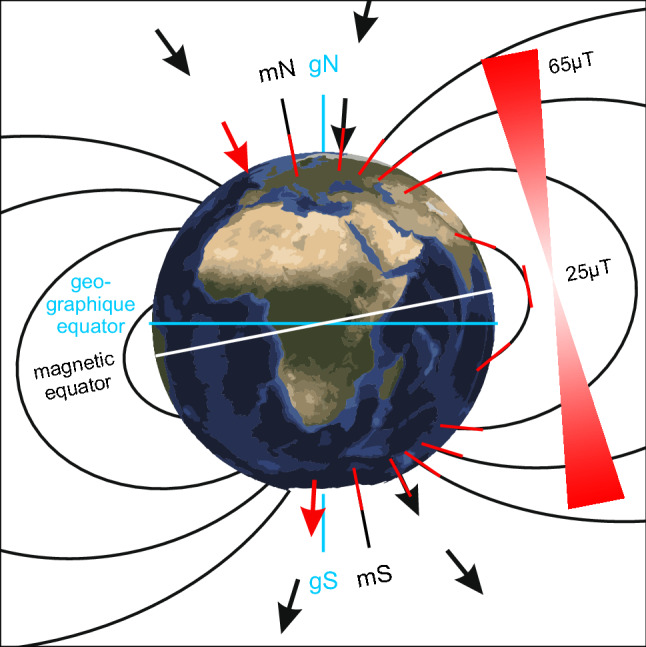
Geomagnetic field (GMF). The GMF offers different cues for navigation: The polarity (red arrows) allows North to be distinguished from South. The magnetic poles (magnetic North mN, magnetic South mS) are shifted relative to the geographic poles (geographic North gN, geographic South gS). The angle between mN and gN is called declination. The intensity (red triangles) is highest at the poles and lowest at the magnetic equator. The inclination is the angle between the GMF lines and gravity (red bars). It changes gradually from the magnetic poles (± 90°) to the magnetic equator (0°)

In principle, the GMF is present always and everywhere on earth making it a promising navigational cue. This contrasts with the fact that magnetoreception is often thought to be a back-up system for navigation in animals. That apparent contradiction makes magnetoreception such a puzzling phenomenon. However, the GMF has different characteristics that can be—and actually are—used by animals for navigational tasks (Clites and Pierce 2017). The GMF encompasses the entire globe, but since the distinct parameters of the GMF vary across the earth in a predictable manner, different cues are available for both compass orientation and position sensing (Fig. 1) (https://www.ngdc.noaa.gov/geomag/faqgeom.shtml). The GMF measured on the surface of the earth can be approximately described by a magnetic field of a magnetic dipole that is tilted with respect to the earth’s rotational axis. The GMF lines originate in the Southern hemisphere and re-enter the earth in the Northern hemisphere. Therefore, the polarity of the magnetic field lines offer a valuable reference system to determine directions almost everywhere on earth, except for the magnetic poles. This information can be used for determining the direction, i.e., as a “magnetic compass”. The angle between the GMF lines and the earth’s surface is called inclination. At the magnetic equator, the inclination is 0°, as the GMF lines are parallel to the earth’s surface. The GMF lines gradually change until the inclination reaches + 90° and − 90° at the magnetic poles, respectively. Additionally, the GMF intensity varies around the globe between 25 µT and 65 µT. Information about inclination and intensity can be combined to infer the position on earth during a journey, i.e., often referred to as a “magnetic map” (e.g., Lohmann et al. 2004). Taken together, the GMF offers a variety of information that could in principle be used by animals for orientation and navigation.

## The magnetic sense

Presently, two main hypotheses of how the GMF might be detected in Hymenoptera have been proposed; the ferromagnetic hypothesis (for a review: Shaw et al. 2015) and the biochemical hypothesis (for a review: Hore and Mouritsen 2016). For both hypotheses, there is empirical evidence (real examples in the animal kingdom), and theoretical evidence, although both mechanisms have advantages and disadvantages. The ferromagnetic hypothesis states that GMF information is first sensed by a sensory neuron that possesses iron-containing molecules mechanically coupled to a sensitive cellular structure, e.g., mechanosensitive ion channels. Most commonly biogenic magnetite is thought to be involved (Shaw et al. 2015; Clites and Pierce 2017). However, titanium has also recently been suggested to be a potential component of the magneto-sensory system in Hymenoptera (Wajnberg et al. 2017). The particle-based mechanism underlying the magnetic compass enables an animal to detect the polarity of the GMF, i.e., to distinguish north from south (Clites and Pierce 2017). In contrast, the biochemical hypothesis based on the radical-pair-mechanism states that the GMF is sensed by a light-dependent biochemical reaction during which electrons are transferred within photoreceptive molecules. This mechanism enables an animal to determine its position with respect to the inclination angle (Clites and Pierce 2017). The most promising candidate for the radical-pair mechanism is cryptochrome as it seems to be crucial for magnetoreception in birds (Hore and Mouritsen 2016), flies (Gegear et al. 2008) and cockroaches (Bazalova et al. 2016). However, Hymenoptera do not possess the light-dependent type of cryptochrome (Yuan et al. 2007). Furthermore, Hymenoptera have been shown to be magnetosensitive in darkness (e.g., ants: Camlitepe and Stradling 1995; Camlitepe et al. 2005; honeybees: Schmitt and Esch 1993). Both indications make a light-dependent mechanism for magnetoreception in Hymenoptera unlikely.

The best known examples of animals that use the GMF are probably migratory animals like birds (for a review: Wiltschko and Wiltschko 2005) or sea turtles (e.g., Lohmann et al. 2004) covering several thousand kilometers during their long journeys. Some indication for a role of the GMF in long-distance migration was also found in insects, in particular in monarch butterflies (Guerra et al. 2014) and bogong moths (Dreyer et al. 2018). However, magnetic fields might also be very helpful for close-range navigation (Wyeth 2010). As we shall see later, the first learning walks of novices in *C. nodus* are a prime example for magnetoreception during close-range navigation.

## Magnetoreception in social Hymenoptera

Before having a closer look at the experiments investigating the GMF as a navigational cue in *A. mellifera* and *C. nodus*, it is worthwhile to start with an overview on what is known about magnetoreception in Hymenoptera in general. There are many bits and pieces, but it is very hard to extract a clear picture from these. To date, many hymenopteran species have been studied, and several turned out to be magnetosensitive under particular conditions or were shown to contain magnetic material in various body parts, often interpreted as a hint for a potential function in magnetoreception (for reviews: Pereira-Bomfim et al. 2016; Vacha 2017; Wajnberg et al. 2010). Although the presence of magnetic materials is highly interesting, whether the animals actually use the GMF as a cue for navigation is a different question that has to be tackled at the level of behavioral experiments, ideally in their natural habitat. Honeybees (*A. mellifera*) and desert ants of the genus *Cataglyphis* are well-studied experimental models for navigation. For that reason, and as the only clear evidence for navigation with the aid of magnetic information in insects comes from these species, in the following we will focus on experiments carried out with them. This further suggests that the GMF is used for navigation in at least two families within the Hymenoptera.

## Magnetoreception in honeybees

As pointed out earlier, the honeybee (*A. mellifera*) was one of the first animal species for which the existence of a magnetic sense has been proposed (Lindauer and Martin 1968). Compared to other hymenopteran species, until today the magnetic sense of honeybees has been the most extensively studied. Honeybees can inform their nest mates about a profitable feeding site by performing waggle dances. During these waggle dances, they encode information about direction and distance of the feeding place visited so that other foragers can find the same spot (von Frisch 1965). The waggle dances on the vertical comb contain a systematic deviation that changes over time called “residual misdirection” (“Missweisung”) (Lindauer and Martin 1968). Importantly, it is dependent on the GMF and disappears when the GMF is eliminated (Lindauer and Martin 1968). Furthermore, when dancing on a horizontal comb (under natural conditions dances are performed on vertical combs) honeybees orient towards the cardinal points of the GMF after some time (Lindauer and Martin 1972; Martin and Lindauer 1977). These early findings have proven that changes of the GMF do have an influence on honeybee behavior. However, the potential benefit for orientation or navigation remained obscure. The first evidence that the GMF may actually be useful for honeybees, though not for navigational purposes, came from the finding that swarms orient their comb building with the GMF (Lindauer and Martin 1972). Further behavioral experiments using an associative conditioning paradigm suggested that honeybees are able to discriminate small differences in magnetic field intensities (Walker and Bitterman 1985, 1989a). Attached magnets on the abdomen disrupted their abilities to discriminate between magnetic field differences (Walker and Bitterman 1989b). In this experiment, honeybees were confronted with a magnetic dipole anomaly (5 cm radius, 350 µT, i.e., almost ten times stronger than the natural GMF (38 µT) at the experimental site). The attached magnets on the abdomen were pieces of stainless-steel wire magnetized by a unidirectional magnetic field pulse (peak intensity: 100,000 µT). These experiments indicate that honeybees can sense magnetic cues under particular circumstances; however, they did not reveal an apparent biological relevance for navigation. Anatomical studies indicate that the magnetic receptor might be in the abdomen, based on the finding of iron-containing cells in that body part (Kuterbach et al. 1982; Kuterbach and Walcott 1986b). Interestingly, the granules found increase in size and number when honeybees get older (Kuterbach and Walcott 1986a; Shaw et al. 2018). The search for the precise location and potential function of a magnetic sensor has not yet been successful in honeybees. Furthermore, it remains unclear for which navigational purposes honeybees might use the GMF under natural conditions.

Only few studies have tested the magnetic field as a potential cue for orientation or navigation in honeybees by actually manipulating the GMF during navigational behavior. One of them (Schmitt and Esch 1993) investigated orientation behavior in walking bees in darkness. The other two (Collett and Baron 1994; Frier et al. 1996) used free-flying honeybees. These experiments are closest to navigation of honeybees under natural conditions. They show that honeybees can use magnetic fields as compass cues to learn a new feeder position (Collett and Baron 1994) and to discriminate patterns in space (Frier et al. 1996).

**Fig. 2:**
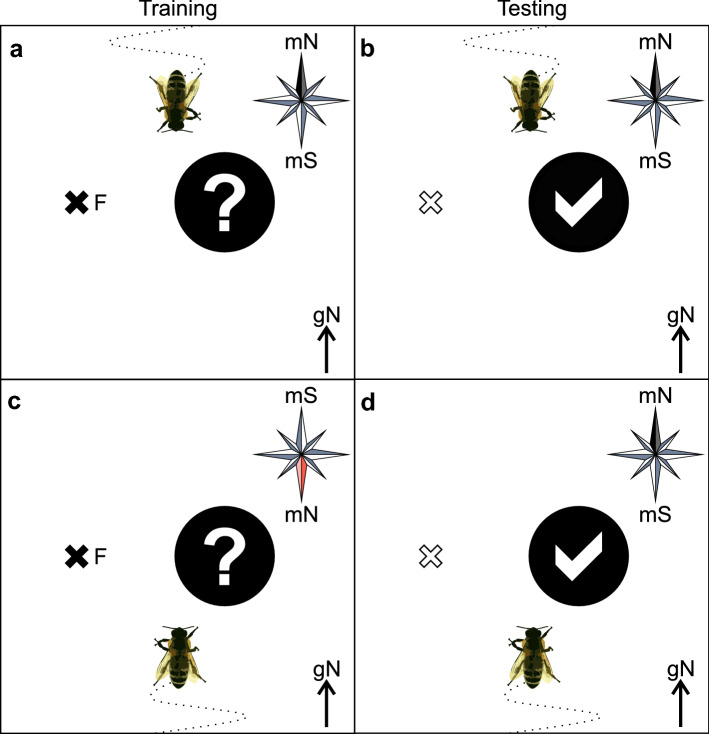
GMF as a compass cue in honeybees. Honeybees were trained to visit a feeder (F, indicated by the black x) placed in a constant compass direction (e.g., west) from a landmark (black circle) within a tent. During **a** training (indicated by the question mark on the landmark) and during (**b)** testing (indicated by the tick on the landmark) when the feeder was removed (indicated by the white x), honeybees faced most often southwards. **c** When the magnetic field was rotated by 180° (magnetic North (mN) pointing to geographic South), honeybees oriented also southwards relative to the experimental magnetic field. **d** When the magnets (i.e., the artificial magnetic field) were removed during testing, honeybees oriented themselves relative to the landmarks and ignored the conflicting information provided by the GMF relative to the panorama. Figure based on data from Collett and Baron (1994)

In the first study (Collett and Baron 1994), honeybees were trained to visit a feeder that had a fixed spatial relationship to a landmark (black cylinder, 3 cm in diameter, 16.5 cm high) positioned 15 cm north, east, south or west of the feeder. Remarkably, during both training (learning the feeder position) and testing (searching for the feeder when it had been removed), honeybees were most often positioned where the feeder had been relative to the landmark (Collett and Baron 1994). Importantly, at this position they most often faced south. By doing so, honeybees occupied a standard viewing direction, and, consequently, kept retinotopic panoramic and compass coordinates in line. When an artificial magnetic field was induced by two rows of permanent magnets on a steel plate, honeybees oriented southwards relative to the experimentally induced magnetic field (strength: 550 µT) (Fig. 2). In contrast, when analyzing their orientations relative to the earth coordinates, bees were randomly distributed. When the magnets were removed after training, trained honeybees nevertheless kept their orientation facing southwards relative to the former experimental magnetic field during the tests (Collett and Baron 1994). The authors concluded that the magnetic field serves as a reference system during learning by guiding the honeybees into their preferred orientation (facing southwards), but the conflict between magnetic information and panorama did not play a role anymore for trained honeybees.

In the second study (Frier et al. 1996), honeybees were trained to discriminate two panoramic patterns that were identical except for their positions in space. To distinguish between the two patterns that were shifted relative to each other by 90°, honeybees must have a reference system informing them about directions. When all directional cues (celestial, panoramic and magnetic) were available, honeybees discriminated between the two patterns easily and chose the correct one significantly more often. In this experiment, honeybees did not need celestial cues to solve the task. When tested in a tent, they still preferred the positive (formerly rewarded) pattern. When the magnetic field was rotated experimentally using permanent magnets (three to seven times GMF strength) or a Merrit coil (GMF strength), honeybees preferentially chose the positive pattern. However, if magnetic and visual cues were in conflict, honeybees relied more on the visual cues than the magnetic cues (Frier et al. 1996). Together, both studies strongly suggest that honeybees can rely on the GMF as a reference system for navigational tasks. However, in both studies honeybees only used the magnetic field when other cues for navigation (especially celestial cues) were absent, indicating that the GMF is not used as the primary navigational cue. This is an important difference for the use of the GMF for navigational purposes between honeybees and desert ants, as will become clear below.

## Magnetoreception in ants

As in honeybees, behavioral and biophysical studies are largely mutually exclusive in ants. The former claim that ants sense the GMF and that magnetic changes influence the ants’ behaviors, whereas the latter aim to identify magnetic particles directly or indirectly in distinct body parts as potential evidence for a magnetic compass (for reviews: Shaw et al. 2015; Wajnberg et al. 2010). The first study that tested the GMF as a navigational cue in ants (*Formica rufa* group) during foraging revealed no influence of magnetic manipulations on the ants’ site allegiance (Ortstreue) (Rosengren and Fortelius 1986). Magnetic sensitivity was first claimed for fire ants (*Solenopsis invicta*) (Anderson and Vander Meer 1993). Their finding, however, was challenged later and could not be reproduced (Klotz et al. 1997). Two species of wood ants (*Formica rufa* and *F. pratensis*) use GMF information during re-visits to a feeder, but only under experimental conditions when all other navigational cues were eliminated (Camlitepe and Stradling 1995; Camlitepe et al. 2005). Leaf-cutter ants (*Atta colombica*) show a behavioral change in walking direction during magnetic manipulations, again when all other cues for orientation are eliminated during foraging (Banks and Srygley 2003; Riveros and Srygley 2008). These results indicate that several ant species are sensitive to magnetic changes. However, in all these studies behavioral changes could be induced only under artificially deprived sensory conditions.

The first experiment in which ants were shown to use magnetic information successfully for a navigational purpose (finding a goal) under semi-natural conditions was performed in desert ants (*C. nodus*). In this case, foragers were trained to return to the nest entrance that was next to a magnet. Indeed, foragers could learn to use such an artificial magnetic landmark as a beacon during nest search (Buehlmann et al. 2012). However, since the field strength was more than 500 times higher than the GMF the use of such a magnetic cue does not prove that the GMF might be used for navigation by the ants. The navigational performances of *Cataglyphis* desert ants have been well known to neuroethologists for decades (for the most recent and extensive review: Wehner 2020). Their abilities to combine celestial compass cues (e.g., the polarization pattern of the sky or the sun’s position, cf. chapter 3 in Wehner 2020, pp. 91ff) with an innate odometer (Wittlinger et al. 2006, 2007) and optic flow (Pfeffer and Wittlinger 2016) to calculate a so-called home-vector, are impressive. During foraging *Cataglyphis* ants constantly keep track of the directions and distances travelled so that they are always informed about their positions relative to the nest (Müller and Wehner 1988). In addition, *Cataglyphis* ants use any cue available for successfully returning to the nest, e.g., visual and olfactory landmarks, the wind or the ground structure (for a review: Wehner 2020).

Importantly, the ants’ navigational system is not fully equipped from the beginning, and the ants have to acquire essential information necessary for navigation as foragers. For that, they perform so-called learning walks, a conspicuous behavior also found in other ant species (for a review: Zeil and Fleischmann 2019). Learning walks are short, explorative trips during which naïve ants—so-called novices—do not collect any food, but acquire information about the nest’s surroundings and calibrate their compass systems (for a review: Grob et al. 2019). At the transition from the dark interior of the nest to outdoor foraging, the novices not only change their behavior drastically, but also undergo substantial neuronal changes in their visual systems attributed to high levels of structural synaptic plasticity (for a review: Rössler 2019). It is crucial for the novices to have enough time and enough space for performing learning walks (Fleischmann et al. 2016, 2018b). Furthermore, learning walks comprise species-specific rotational elements, i.e., so-called voltes and pirouettes (Fleischmann et al. 2017). Voltes are full, walked circles without any directed stops. The function of voltes is not yet known, but we hypothesize that they are crucial for calibrating the celestial compass. In contrast, pirouettes are full or partial turns about the ant’s body axis during which the ants frequently stop. During the longest stopping phase, the gaze direction is precisely and accurately directed toward the nest entrance. Interestingly, only those ant species that inhabit a cluttered environment include pirouettes in their learning walks (Fleischmann et al. 2017). We assume that during learning-walk pirouettes the ants take visual “snapshots” of their homing direction to know where to go when returning from a far-ranging foraging trip. The goal is the nest entrance, only a tiny hole in the ground, practically invisible from the ant’s perspective. It has been suggested before that desert ants use the path integrator based on celestial compass information to take snapshots of the nest’s surroundings (Graham et al. 2010; Müller and Wehner 2010). Since *Cataglyphis* ants heavily rely on celestial cues for path integration during foraging, we expected that they also use celestial cues to align their gaze directions during learning-walk pirouettes. However, surprisingly, that is not the case—*Cataglyphis* novices neither need a direct view of the sun, nor the celestial polarization pattern in the UV-spectrum, to look back to the nest entrance (Grob et al. 2017). Instead, experiments revealed very clear evidence that the ants use the GMF for aligning their gaze directions during their initial learning-walks (Fleischmann et al. 2018a). This was the first proof of an insect using the GMF for a navigational purpose, specifically close-range path integration.

**Fig. 3:**
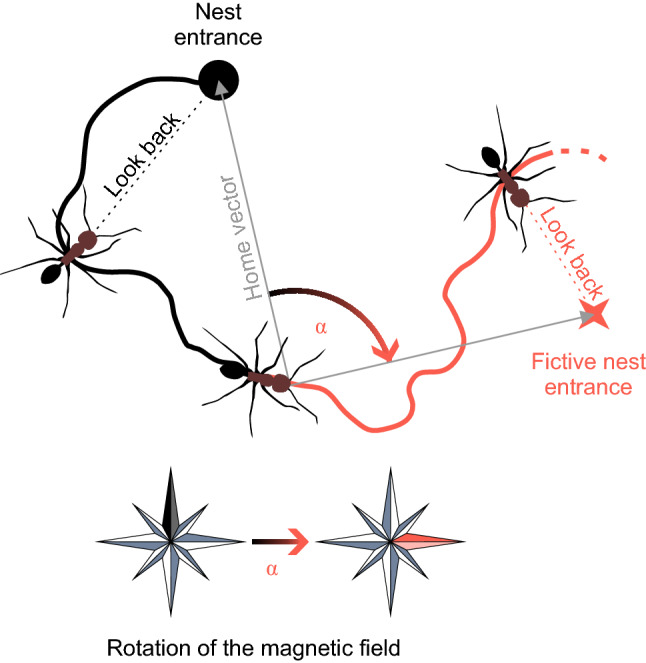
GMF as a compass cue in desert ants. A novice (*C. nodus*) leaves the nest entrance (black circle) and performs a learning walk (black solid line). During the longest stopping phase of a pirouette, it looks to the nest entrance (black dotted line). When the horizontal component of the GMF is rotated (e.g., 90°) the home vector of the ant points towards a new, fictive position of the nest entrance (red star). The novice continues its path (red solid line). During the longest stopping phase of a subsequent pirouette, it looks to the fictive nest entrance (red dotted line). From Fleischmann et al. (2018a)

To test what reference system *C. nodus* novices use for aligning their gaze directions during learning-walk pirouettes, different potential cues for navigation were manipulated at the nest entrance. Under natural conditions, i.e., when both celestial and magnetic cues were available without any changes, the novices reliably looked back to the nest entrance during the longest stopping phase of a pirouette (Fleischmann et al. 2017, 2018a; Grob et al. 2017). Different visual filters were installed above the nest entrance, but the novices always accurately and precisely looked back to the nest entrance (Grob et al. 2017). This shows that celestial cues were not necessary for the ants to accomplish this navigational task. When an electromagnetic spiral was set up around the nest entrance that disarrayed the GMF completely, the gaze directions of the novices were randomly distributed (Fleischmann et al. 2018a). This experiment provided initial evidence that *C. nodus* novices use the GMF as compass cue during learning walks. Further support for this hypothesis came from experiments that were performed with a Helmholtz coil. Elimination of the horizontal component of the GMF also led to randomly distributed gaze directions (Fleischmann et al. 2018a). Furthermore, by rotating the horizontal component by either 180° or ± 90° the gaze directions of the ants were rotated to the fictive position of the nest entrance (Fleischmann et al. 2018a). That means that the ants actually use magnetic information and feed it into their path integrator (Fig. 3). Therefore, the GMF is the necessary and sufficient compass cue to align the gaze directions during learning walk-pirouettes in *C. nodus*. Until now, this is the only evidence that a hymenopteran insect—or any insect—relies exclusively on the GMF for performing a navigational task under natural conditions. *C. nodus* novices do not use the magnetic compass as a subordinate or back-up cue, rather the GMF is the decisive reference system during initial learning walks.

## Future prospects: investigation of the hymenopteran magnetic compass in *Cataglyphis*

The recently discovered magnetic compass in the desert ant *C. nodus* opens up new possibilities for investigations aimed at understanding the nature of the hymenopteran magnetic compass. The learning-walk pirouettes performed by *Cataglyphis* novices provide an ideal behavioral read-out to investigate the underlying magnetic compass in more detail. Furthermore, the magnetic compass used for this navigational task is a unique example of an essential magnetic compass used for close-range navigation. Wyeth (2010) suggested that using a magnetic compass for navigation over short distances might be beneficial under certain circumstances. The *Cataglyphis* example described here meets all four criteria brought up by Wyeth (2010): First, using the primary cue—which would be celestial cues—is not feasible, because naïve ants have to perform the learning walks to acquire all visual information necessary to calibrate their compass systems for visually based navigation as foragers. Second, the nest entrance (goal) does not move. Third, the novices usually do not drift during learning walks. Fourth, other spatial representations of the environment—which might be for example information about the panorama around the nest—are not yet available and, therefore, constrained. For that reason, the magnetic compass of *Cataglyphis* might have a biological importance equal to that of animal species that are well known magnetosensitive navigators pursuing completely different navigational tasks like migration over long distances.

Several research topics need to be tackled in the future to understand the magnetic sense of Hymenoptera. The corresponding key questions are:

- What are the characteristics of the hymenopteran magnetic compass?
- Where are the magnetic sensors located?
- Is magnetoreception in *Cataglyphis* ants during learning walks an active sensing process?
- How is magnetic information processed in the brain and how is it used for navigation?
- Why do the animals rely on the GMF only under certain conditions, but not under others?

**Fig. 4:**
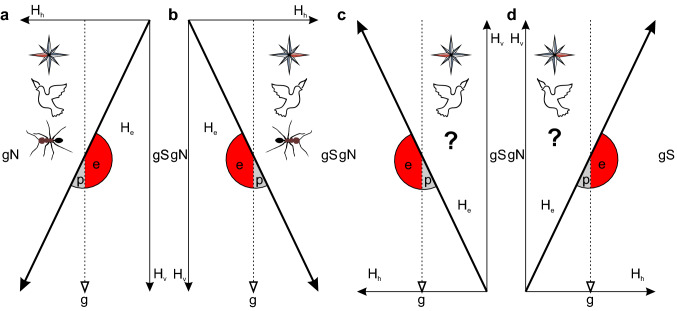
Differentiation between polarity and inclination compass. Under different experimental conditions, a polarity compass and an inclination compass will lead to different outcomes. **a** Natural magnetic field (H_e_) in the northern hemisphere that is composed of the horizontal component (H_h_) and the vertical component (H_v_). **b** Horizontal component reversed. **c** Vertical component reversed. **d** Both horizontal and vertical component reversed. A polarity compass points towards magnetic north, i.e., in the direction of H_h_. In contrast, an inclination compass measures the angle between the GMF lines and gravity (g), and distinguishes between poleward (p) and equatorward (e). Several bird species possess an inclination compass. For desert ants, it is an open question whether they possess a polarity compass or an inclination compass. Figure adapted from (Wiltschko and Wiltschko 2005; Lambinet et al. 2017)

To answer the first questions, the biological nature of the magnetic receptor needs to be investigated. We hypothesize that *Cataglyphis’* magnetic compass is light-independent, polarity-sensitive, and, therefore, most likely particle-based. Since *Cataglyphis* ants do not perform learning-walk pirouettes in complete darkness, another navigational task needs to be found to test whether the ants can use the GMF in complete darkness. However, since several Hymenoptera have already been shown to be magnetosensitive in darkness (e.g., Camlitepe and Stradling 1995; Camlitepe et al. 2005; Schmitt and Esch 1993), this is most likely to be true also for *Cataglyphis* ants. The conclusive experiment to test whether *Cataglyphis* actually has a polarity-sensitive compass has yet to be performed. For that, the vertical and the horizontal component of the GMF have to be manipulated separately using 3D Helmholtz coils (Fig. 4). For honeybees it has already been shown that their magnetic compass is polarity-sensitive under laboratory conditions (Lambinet et al. 2017). Further evidence for a particle-based mechanism might come from magnetic pulse-experiments as have been performed in other arthropods (e.g., spiney lobsters: Ernst and Lohmann 2016). If the results of these behavioral experiments reveal the nature of the hymenopteran magnetic compass, the search for the magnetic sensors will be most promising. Another open question is whether the magnetic compass in *Cataglyphis* is biomineralized or whether the animals obtain the magnetic particles from the environment. In leaf-cutter ants, it has been shown, that only individuals that had contact to the natural soil were able to respond to changes in the GMF, while lab-reared leaf-cutter ants where unable to do so (Riveros et al. 2014).

Understanding the characteristics of the magnetic compass will help to answer the questions of the second topic about the mechanisms underlying magnetoreception in Hymenoptera. In principle, the magnetic sensors can be located anywhere in the body. However, since the magnetic sensors have to be linked to the nervous system, it appears more likely that they are embedded in a periphal sensory structure or system. A number of studies were already aimed to find evidence for particle-based magnetic compasses in Hymenoptera, for example using magnetic-based techniques like superconducting quantum interference device (SQUID), magnetometry, or magnetic resonance by analyzing the entire animal (for a review: Shaw et al. 2015). Based on the amount of magnetic material, the respective authors suggested, where the magnetic compass might be located. In honeybees, the abdomen has attracted a lot of attention (e.g., Hsu and Li 1994), but, at the same time, received serious criticism (Nichol et al. 1995). It has remained an open question whether the honeybee abdomen actually plays a crucial role in magnetoreception or has a function as waste storage for dietary iron (Shaw et al. 2018). Based on the studies offering indirect evidence for the location of the magnetic compass, several studies have pointed at the hymenopteran antenna as a potential location, particularly in ants (Abraçado et al. 2008; de Oliveira et al. 2010; Wajnberg et al. 2017) and stingless bees (Lucano et al. 2006). Even though the mere existence of ferromagnetic material somewhere in the animal does not prove a magneto-sensitive organ, the hymenopteran antenna is a promising candidate for the magnetic compass. Within the antenna resides the Johnston’s organ, a multimodal mechanosensitive sensory organ that was shown to serve the detection of antennal vibrations or deflections caused by gravity, wind or touch. In honeybees it was suggested to sense antennal deflections caused by electric fields (Greggers et al. 2013). Conceivably, given its circular organization (Vowles 1954; Ai et al. 2007), the Johnston’s organ could also be sensitive in some way to the GMF. A first piece of evidence supporting this hypothesis might have already come from the very first experiments with honeybees. The systematic error (*Missweisung*) in the waggle dance indicate that changes dependent on the GMF—and disappear when the GMF is eliminated (Lindauer and Martin 1968)—might result from a sensory conflict. Gravity is sensed with the Johnston’s organ (Vowles 1954). If the Johnston’s organ also receives input from the GMF, the information about gravity and about the GMF might become mixed up and can no longer be disentangled completely from each other resulting in the *Missweisung*. Another indication comes from the behavior of the ants. *Cataglyphis* novices erect their antennae conspicuously during their first learning walks (Wehner et al. 2004). This may suggest that specific antennal movements and postures are necessary to receive GMF information in an active sensing process. Active sensing processes are neuronal processes in which not only external changes of the sensory stimuli are taken into account, but also motor acts by the sensory organ.

Not only are the magnetic receptors unknown, but also the neuronal pathway along which the magnetic information is processed. Since manipulation experiments have shown that *C. nodus* novices integrate magnetic information into their path integrator, magnetic information, most likely, feeds into the central complex (CX). The CX is a multisensory integration center in the middle of the insect brain processing information important for sensory orientation and navigation (for a review: Pfeiffer and Homberg 2014) and comprises neuronal circuits of insect path integration (Stone et al. 2017). For example, the celestial polarization pattern is topographically represented in components of the CX, very much like a compass (for a review: Pfeiffer and Homberg 2014). The GMF information should be represented likewise. Only when all navigational information converges in this neuronal integration center, a particular travel direction can be decided and relayed as motor output (Grob et al. 2019). However, it is also conceivable that magnetic information might be relayed onto pathways up- or downstream to the CX. Recently, a detailed 3D-atlas of neuropils and connecting tracts in the *Cataglyphis* brain has been published (Habenstein et al. 2020). This greatly enhances the possibility to track potential connections from sensory structures into individual neuropils in the central brain in unprecedented detail. During the transition from interior worker to exterior forager, the brain of *Cataglyphis* ants undergoes plastic changes in synaptic circuits associated with the visual system (for a review: Rössler 2019). There may as well be additional changes in neuronal circuits associated with the magnetic sense resulting in a different synaptic weight that GMF sensory information might have at different life stages from inside the nest to the period of naïve learning at the beginning of the foraging career and/or later on during foraging. These neuronal dynamics combined with careful investigations of the conditions under which Hymenoptera rely on the GMF for navigation may help to pin down the nature of the magnetic compass and its central pathways in the brain. Strikingly, honeybees rely on magnetic information when learning a feeding place, but after gaining enough experience, i.e., after acquisition of the panorama, they do not show any changes in behavior when the magnetic and other navigational information are in conflict (Collett and Baron 1994). Since until now the use of a magnetic compass in *Cataglyphis* ants has only been shown in novices, it will be crucial to see whether *C. nodus* ants also rely on the GMF during re-learning walks, or whether they then use other navigational cues like (the already established) celestial compass or the surrounding (known) landmark panorama. Re-learning walks can be induced in experienced foragers by changing the panorama around the nest entrance (Fleischmann et al. 2016). If evidence for an experience-independent magnetic compass can be found, further experiments in the laboratory will be possible, even when often only non-naïve animals are available. All these future prospects make *Cataglyphis* ants a highly promising experimental model for a truly integrative approach aiming at understanding the molecular and cellular mechanism of magnetoreception, the function and plasticity of the associated neuronal circuits, all the way up to the behavioral implications underlying a magnetic compass used for animal navigation.
